# Pericyclases for cycloaddition

**DOI:** 10.1093/nsr/nwac229

**Published:** 2022-10-26

**Authors:** Bo Zhang, Hui Ming Ge

**Affiliations:** State Key Laboratory of Pharmaceutical Biotechnology, Institute of Functional Biomolecules, School of Life Sciences, Chemistry and Biomedicine Innovation Center (ChemBIC), Nanjing University, China; State Key Laboratory of Pharmaceutical Biotechnology, Institute of Functional Biomolecules, School of Life Sciences, Chemistry and Biomedicine Innovation Center (ChemBIC), Nanjing University, China

## Abstract

Focusing on the cycloadditions, which have been widely utilized in total synthesis, this perspective reviews the flourish research of pericyclase for cycloaddition and discusses existing challenges.

The Diels–Alder (DA) reaction is a simple yet very powerful pericyclic transformation involving the cycloaddition of a 1,3-diene and a dienophile to synthesize a cyclohexene ring via a cyclic transition state. Its importance was recognized by Nobel Prizes in Chemistry. Since the first application of the DA reaction in the total synthesis of the steroids cortisone and cholesterol in 1952, significant progress has been made in the understanding and utility of DA reactions [1]. In particular, enzyme-catalysed DA reactions participate in the biosynthesis of numerous cyclohexene-containing natural products. However, although enzymatic pericyclic reactions such as the Claisen rearrangement catalysed by chorismate mutase have been known since 1984, the prevalence of pericyclases in biosynthesis remains unclear. This perspective presents the research progress on enzymatic cycloaddition reactions and outlooks the challenges and prospects in the study of pericyclases.

In the 1990s and 2000s, the enzymes macrophomate synthase, lovastatin polyketide synthase, solanapyrone synthase and riboflavin synthase were identified and proven to catalyse cyclohexene ring formation. However, their role as real DAases was not clear because they work in a non-concerted or multifunctional manner [2]. In 2011, H. Liu *et al.* characterized SpnF as the first dedicated DAase, which can specifically accelerate the DA reaction of a macrolactone substrate to form a 5/6/15-tricyclic intermediate in the biosynthesis of insecticide spinosyn A through a single ambimodal transition state [3] (Fig. 1A). In 2015, W. Liu and co-workers reported PyrE3 and PyrI4 as DAases that act in tandem to form two cyclohexene rings in pyrroindomycin biosynthesis (Fig. 1B). Both cycloadditions are enzyme-dependent and proceed regio- and stereoselectively to give an enantiomerically pure pentacyclic scaffold. The discovery of these dedicated DAases together with the advance of genome sequencing has boosted the identification of diverse DAases. For example, the homologous proteins of PyrE3 and PyrI4 were identified in the biosynthesis of abyssomicin, kijanimicin, chlorothricin and versipelostatin, and the fungal DAases Fsa2, CghA, MycB and PvhB were reported to build decalin rings in equistetin, sch210972, myceliothermophin E and varicidin A, respectively. After these enzymes were reported, the term ‘pericyclase’ was coined by the groups of Tang and Houk [4].

**Figure 1.**
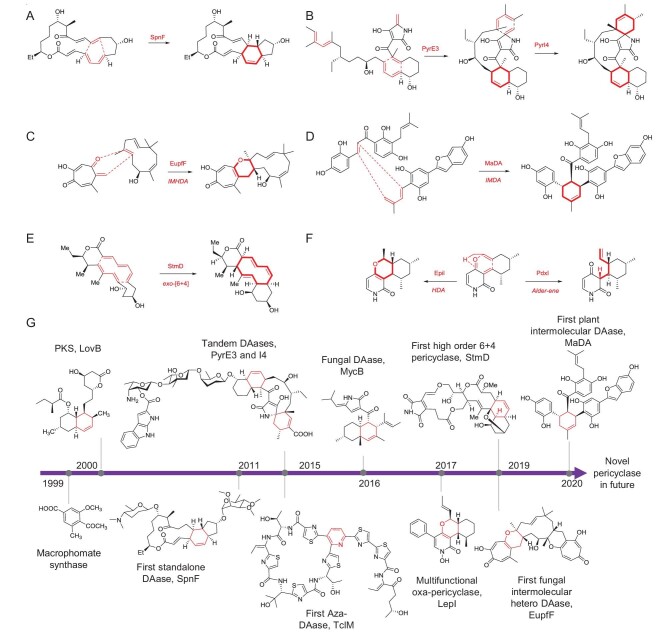
Selected pericyclases. (A) SpnF, (B) PyrE3 and PyrI4, (C) EupfF, (D) MaDA, (E) StmD, (F) EpiI and PdxI; (G) timeline of the advances in the investigation of pericyclases.

In contrast to the numerous intramolecular DAases, other types of enzymatic pericyclic reactions are very rare with only a few examples. TclM catalyses an oxa-DA reaction to forge the trisubstituted pyridine core of thiocillin. Hu *et al.* characterized EupfF as the first fungal intermolecular hetero-DAase [5] (Fig. 1C). Shortly after, Lei *et al.* identified the flavin adenine dinucleotide-dependent enzyme MaDA that can catalyse an intermolecular DA reaction to produce the isoprenylated flavonoid chalcomoracin in plant [6] (Fig. 1D). Our group discovered a group of enzymes that catalyse [6 + 4] and [4 + 2] cycloadditions through a single ambimodal transition state in the biosynthesis of streptoseomycin-type compounds [7] (Fig. 1E). This constitutes the first directly observed and verified enzymatic high-order cycloaddition since its prediction by Woodward and Hoffmann in 1965. Tang *et al.* identified LepI as a multifunctional S-adenosylmethionine-dependent pericyclase that catalyses three pericyclic reactions including DA, oxa-DA and retro-Claisen rearrangement in the biosynthesis of a fungal metabolite leporin [8]. Inspired by the discovery of LepI and the characteristic vinylcyclohexene core in pyridoxatin-type natural products, Tang further identified two homologous groups of enzymes, which catalyse two distinct pericyclic reactions: an Alder-ene reaction and an oxa-DA reaction, respectively [9] (Fig. 1F). The discovery of these enzymes significantly expanded the catalytic diversity of pericyclases and provided access to new enzymatic tools for the efficient synthesis of natural products.

Pericyclases have been extensively studied for >10 years. However, many questions remain unsolved. The main challenges regarding these enzymes can be summarized as follows:

- The identified pericyclases are just the tip of the iceberg. Pericyclases are phylogenetically and structurally distinct and evolved independently from diverse ancestors including oxidoreductases, dehydratases, hydratases, methyltransferases and regulators. Thus, identifying pericyclases by sequence alignment or 3D structure prediction is difficult. Many of the encoded enzymes have no characterized homologs and the accumulated metabolites from the gene mutant strains are often non-enzymatically cyclized, hindering the identification of pericyclases. For example, malbrancheamide and brevianamide are fungal natural products with similar skeletons; however, the former is biosynthesized via an enzymatic DA reaction, whereas a non-enzymatic DA reaction affords the latter.
- The mechanism of action of pericyclases remains unclear. Some pericyclases act as entropic traps that accommodate and activate the corresponding substrate into a reactive preorganized state, which readily undergoes pericyclization. However, the experimental study of enzymatic transition states is challenging, which hinders the mechanistic characterization of putative pericyclases. Thus, much of the work done on the mechanisms of these enzymes has been computational in nature and more experimental investigations are essential.
- Further advances in the bioengineering of pericyclases are required. The pericyclases identified so far show strict substrate preference and their activity is highly dependent on the corresponding substrate. However, significant breakthroughs in pericyclase bioengineering have been recently achieved. In the original study of the enzymatic Alder-ene reaction, the authors partially altered the pericyclic reactions by changing one or two residues located in the active cave of the pericyclase using the crystal structure [8]. Similarly, after solving the crystal structure of a CghA–product complex, Watanabe reversed the diastereoselectivity of CghA from strictly *endo*-selective to the energetically disfavored *exo*-selective by simply changing three key residues [10]. Furthermore, the substrate promiscuity of MaDA allowed Lei to use it as a catalytic tool to achieve the first chemoenzymatic total synthesis of artonin I.

Progress in big genomic data and bioinformatics will revolutionize natural product biosynthesis and unveil new pericyclases. Structure biology, biochemistry and computational studies will allow reprogramming pericyclases to produce high-value products. The design of artificial pericyclases and their application in synthetic chemistry and synthetic biology are expected.
